# Insight into the ERVK Integrase – Propensity for DNA Damage

**DOI:** 10.3389/fmicb.2016.01941

**Published:** 2016-12-01

**Authors:** Samantha Bray, Matthew Turnbull, Sherry Hebert, Renée N. Douville

**Affiliations:** ^1^Douville Lab, Department of Biology, University of Winnipeg, WinnipegMB, Canada; ^2^Department of Immunology, University of Manitoba, WinnipegMB, Canada

**Keywords:** endogenous retrovirus-K, integrase, DNA damage, genomic instability, neurological disease, cancer

## Abstract

Retroviruses create permanently integrated proviruses that exist in the host genome. Retroviral genomes encode for functionally conserved *gag, pro, pol*, and *env* regions, as well as integrase (IN), which is required for retroviral integration. IN mediates viral genome insertion through 3′ end processing of the viral DNA and the strand transfer reaction. This process requires the formation of a pre-integration complex, comprised of IN, viral DNA, and cellular proteins. Viral insertion causes DNA damage, leading to the requirement of host DNA repair mechanisms. Therefore, a failure of DNA repair pathways may result in genomic instability and potentially cause host cell death. Considering the numerous human diseases associated with genomic instability, the endogenous retrovirus-K (ERVK) IN should be considered as a putative contributor to DNA damage in human cells. Future research and drug discovery should focus on ERVK IN activity and its role in human conditions, such as neurological disease and cancers.

## Introduction

Retroviruses have mastered the art of horizontal gene transfer. A key viral enzyme in this process is the retroviral integrase (IN) enzyme which catalyzes the merger of viral and host genomes. Starting from an RNA genome, retroviruses convert their genetic material into double stranded DNA (dsDNA) using a virally encoded reverse transcriptase (RT) enzyme. The viral dsDNA is then transported into the nucleus as part of the pre-integration complex (PIC), which is composed of both viral and host proteins, including IN (reviewed in Jayappa et al., 2012; Gerard et al., 2013). IN coordinates processing of the linear viral DNA ends and joining those ends into target host DNA through a strand transfer reaction (reviewed in Lesbats et al., 2016). Subsequently, DNA lesions are left in the host genome, which require cellular repair mechanisms to restore genomic integrity. Aberrant IN activity or imperfect repair mechanisms can leave a host vulnerable to genomic instability through the accumulation of DNA lesions. This paper provides a perspective on how the endogenous retrovirus-K (ERVK) IN enzyme may play a role in generating genomic instability in the context of human disease.

### Structure of Retroviral Integrases

**FIGURE 1:**
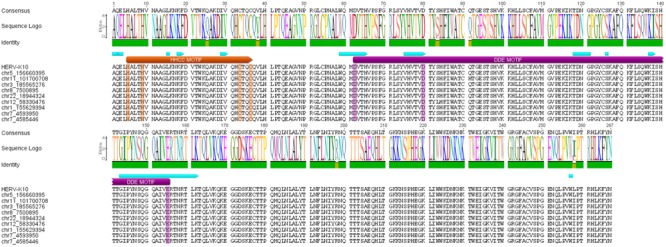
**The ERVK integrase (IN) contains conserved motifs required for enzymatic activity.** The alignment of ERVK HML-2 IN sequences with intact open reading frames. Apart from an intact ERVK-10 (chr5 156660395, 5q33.3) (Kitamura et al., 1996), several other genomic loci containing IN were identified and labeled based on chromosome, position and cytological band: ERVK7 on chr1 155629394, 1q22; ERVK11 on chr3 185565276, 3q27.2; ERVK6b on chr7 4593950, 7p22.1; ERVK6a on chr7 4585446, 7p22.1; ERVK8 on chr8 7500895, 8p23.1; ERVK25 on chr11 101700708, 11q22.1; ERVK21 on chr12 58330476, 12q14.1; ERVK24 on chr22 18944324, 22q11.21. All chromosomal locations are reflective of the human genome (GRCh38) assembly. The HHCC and DDE motifs present in the IN enzyme have been annotated in orange and purple, respectively. Blue annotations reflect additional amino acids conserved within retroviral IN proteins.

The structure of a retroviral IN commonly contains three domains which are the N-terminal domain (NTD), the central catalytic domain (CCD), and the C-terminal domain (CTD); some also encode an additional N-terminal extension domain (NED) (reviewed in Lesbats et al., 2016). The NTD is involved in IN multimerization and contains two conserved histidine (H) and cysteine (C) residues that form a highly conserved zinc-binding HHCC motif found in all retroviral IN (**Figure 1**) (reviewed in Zheng et al., 1996; Lesbats et al., 2016). The CCD contains two highly conserved aspartic acid (D) residues and a glutamic acid (E) residue that form a catalytic triad called the DDE motif (Hare et al., 2010). This catalytic triad recognizes and binds to Mg^2+^ which is essential for proper IN function and multimerization (reviewed in Lesbats et al., 2016). The acidic residues of the DDE motif catalyze 3′ linear DNA processing, DNA strand transfer reactions, and disintegration reactions (reverse of the strand transfer ligation reaction) – all enzymatic processes required for a functional IN (Hossain et al., 2013; Wolkowicz et al., 2014). The CTD is the least conserved domain, although it contains some conserved tryptophan residues, and is integral to the formation of the intasome (reviewed in Lesbats et al., 2016). Post-translational modification of IN enzymes can also impact their activity. For HIV, acetylation of lysine residues within the CTD by cellular proteins, p300 and GCN5, enhances the DNA binding capacity and strand-transfer activity of IN (Cereseto et al., 2005; Suzuki and Chew, 2012). Additionally, integrase enzymes function in higher-order multimeric complexes, as determined by protein analysis and crystallography (Diamond and Bushman, 2005; Li et al., 2011).

Integrase carries out important enzymatic functions that integrate reverse-transcribed viral DNA into host cell DNA. However, IN does not function alone, as the functional PIC also contains host proteins. In HIV and Moloney murine leukemia virus (MoMLV), IN is associated with barrier-to-autointegration factor (BAF), which helps prevent integration of the viral DNA into itself (Van Maele et al., 2006; Wiebe and Jamin, 2016). It would be of great interest to determine whether BAF is a universally utilized mechanism by which retroviruses are protected from autointegration, or whether divergent mechanisms have evolved. Retroviruses also utilize host cell proteins in the identification of suitable integration sites (reviewed in Kvaratskhelia et al., 2014). Lens epithelium-derived growth factor (LEDGF) directs HIV IN to target integration sequences and protects IN from proteolysis (Gerard et al., 2013). More recently, the bromo- and extra-terminal domain (BET) proteins have been demonstrated to function in proviral targeting in MoMLV (De Rijck et al., 2013). The varied preferences exhibited for retroviral insertion suggests that ERV targeting will also utilize unique cellular proteins.

### Integrase Function and the Consequences of Failed DNA Lesion Repair

Integration is carried out in three steps: (i) processing, (ii) joining, and (iii) host-mediated DNA repair (reviewed in Lesbats et al., 2016). In the first step of retroviral DNA integration, IN binds to the linear viral dsDNA to form a stable complex called an intasome. IN then cleaves two (or three) nucleotides from the 3′ ends of viral dsDNA to produce 3′ hydroxyl groups. Next, IN coordinates the cleavage of host DNA phosphodiester bonds and their ligation to the free viral hydroxyl groups. Retroviral integration is not a perfect process. The strand transfer complex (STC) leaves behind single-stranded DNA (ssDNA) gaps at the host-provirus junctures and two 5′ base pair overhang extensions of the viral DNA. Cellular DNA repair mechanisms are then required to restore genome integrity (Yoder and Bushman, 2000; reviewed in Lesbats et al., 2016). However, the process by which the STC is disassembled to allow for DNA repair remains unknown.

Accumulation of DNA lesions and genomic instability lead to loss of cellular functionality, and ultimately the death or transformation of human cells (Hanahan and Weinberg, 2011). Unrepaired IN-mediated ssDNA lesions are hotspots for the formation of double stranded breaks (DSB) in the DNA of replicating host cells (Ryan et al., 2016). Products of IN activity such as (i) newly integrated proviruses, (ii) viral episomes resulting from autointegration (2LTR loops), and (iii) unresolved lesions in the host genome may signal the host cell to initiate an innate immune response and definitely signal engagement of diverse DNA damage repair pathways critical to successful integration; failure to repair the ssDNA lesions left by IN can induce apoptosis (Stetson et al., 2008; Bregnard et al., 2014; Ryan et al., 2016).

Interestingly, DSBs can enhance the integration of HIV DNA into the host genome when in the presence of catalytically inactive IN (Ebina et al., 2012; Koyama et al., 2013). Complementary findings that IN is dispensable for provirus insertion in the context of DSBs stem from the observation of enhanced integration of wild-type IN virus when DNA-damaged cells were treated with the IN inhibitor raltegravir (Koyama et al., 2013). This study suggests that IN inhibitors are best suited to prevent DNA damage in healthy cells, and that both inhibition of RT (formation of viral DNA) and IN would be required in cells containing DNA lesions.

### Mobile Elements in the Human Genome as a Source of Genomic Instability

The human genome contains many endogenous retroviruses which potentially encode IN (Seifarth et al., 2005). Additionally, enzymes encoded by non-LTR retrotransposons are known to mediate DNA damage in humans (e.g., ORF2p) (Gasior et al., 2006). Select elements, such as ERVK and long interspersed nuclear element-1 (LINE-1) are of special interest because of their retrotranspositional activity in modern humans (Mills et al., 2007). For example, both ERVs and LINE-1 are active in the developing human brain, and then repressed in mature tissues (reviewed in Erwin et al., 2014; Mortelmans et al., 2016). Both ERVK and LINE-1 are negatively regulated by members of the APOBEC3 family (Lee et al., 2008; Jones et al., 2013), and differential activity of these elements has been independently linked to human disease (Frank et al., 2005; Douville et al., 2011; Balestrieri et al., 2014; Bundo et al., 2014). Therefore, it is possible that these mobile elements generate similar patterns of genomic instability when active. Although this paper calls attention to the underappreciated role of ERVK IN, it is not the only endogenous enzyme which may induce DNA damage and subsequent genomic instability.

### ERVK Encodes a Functional Integrase

The original description of a functional IN in ERVK-10 (HERV-K10) was by Kitamura et al. (1996). This retroviral enzyme shows not only terminal cleavage and strand transfer activities of ERVK LTR substrates, but also of LTRs from the divergent retroviruses, HIV and RSV (Kitamura et al., 1996). Despite these findings, there has been an underwhelming interest in ERVK IN and their potential role in human biology.

Recently, our team sought to determine if additional ERVK loci related to ERVK-10 (those in the HML-2 clade) encode potentially active IN enzymes. IN sequences were identified in the human genome GRCh38 by a tBLASTn search based on the region of the reconstituted infectious ERVK virus *Pheonix* (Dewannieux et al., 2006) which matches the Pfam entries for each IN domain: NTD (Integrase_Zn, PF02022), CCD (rve, PF00665), CTD (IN_DBD_C, PF00552). BLAST hits from the same ERV were merged and extended according to their alignment by MACSE. Out of the 20 ERVK proviruses containing a full-length IN ORF, only nine maintained intact HHCC and DDE active site motifs (**Figure 1**). All putative ERVK IN-encoding ORFs contained signature HALTH (HXXXH) and CTQC (CXXC) sequences for the HHCC motif. Additionally, all but one of these sequences included a WQMD signature associated with the first aspartate, and a TDNG signature was consistently associated with the second aspartate within the D(X_17_)D(X_35_)E motif.

Select mutations within the HIV IN have been shown to modulate its catalytic activity (class I mutants) (Engelman, 1999). When comparing amino acid substitutions within mutant HIV IN enzymes and the consensus ERVK IN sequence, we found that ERVK IN contains no known inactivating substitutions that alter the active sites or the activity of 3′ processing, DNA binding or joining (Konsavage et al., 2007; Li et al., 2012; Johnson et al., 2013). However, without a clear understanding of ERVK IN and cellular protein interaction, it is difficult to interpret which substitutions would impact PIC formation and STC disassembly. Further, ERVK IN likely plays a role in processes other than integration, such as enhancement of reverse transcription and virion assembly (reviewed in Lesbats et al., 2016).

**FIGURE 2:**
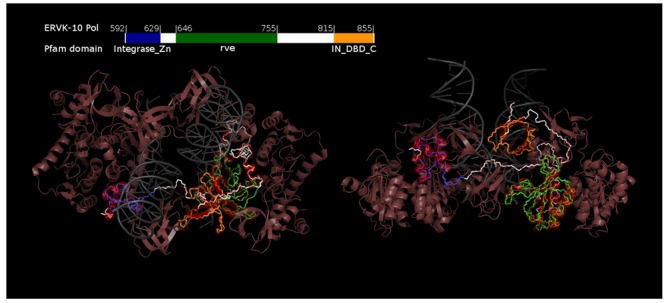
**Homology model of the ERVK-10 integrase.** The ERVK-10 IN (Uniprot P10266) was modeled by homology to the betaretrovirus Mouse Mammary Tumor Virus (MMTV) intasome (PDB 3JCA). 100 models were produced using Modeller 9.15 using default settings except the maximum distance for inter-alpha-carbon homology derived constraints was 32 Angstroms and the slow default was used for molecular dynamics. The model with the lowest DOPE score (-26429) was superimposed onto the template chain (3JCA:A) by their alpha carbon co-ordinates in pymol, and the RMSD between all 260 aligned alpha carbons is 0.275 Å. ERVK backbone carbons and nitrogens are colored so regions matching the Pfam entries Integrase_Zn, rve, and IN_DBD_C match the color of their linear representation on the ERVK-10 sequence. 3JCA is light red except the template chain is bright red, and the DNA is gray. This figure was produced using Pymol and GIMP.

A model of a single ERVK IN subunit superimposed on the Mouse Mammary Tumor Virus (MMTV) intasome (**Figure 2**) shows that the predicted protein folding results in the expected domain architecture for betaretroviral IN (Ballandras-Colas et al., 2016). Moreover, the orientation of key residues forms a clear active site conformation. The DNA binding affinity of the ERVK IN may differ from MMTV IN because of a linker between the NTD and CCD domains that impinges into the DNA binding site, and may partially explain the more relaxed substrate specificity of the ERVK IN toward retroviral LTRs (Kitamura et al., 1996). Without a validated ERVK intasome model, it will be difficult to predict mutations that may impact multimerization and drug resistance (reviewed in Lesbats et al., 2016).

### ERVK-Associated Diseases Are Associated with Genomic Instability

The expression of ERVK IN in human cells is poorly documented. However, cortical brain tissue from patients with amyotrophic lateral sclerosis (ALS) contain an enhanced frequency of ERVK transcripts from proviral loci containing open reading frames (ORFs) for IN, when compared to controls with systemic disease (Douville et al., 2011). Moreover, the presence of functional RT expression in several disease states, including ALS (Douville et al., 2011) and breast cancer (Golan et al., 2008), alludes to the possibility of IN co-expression derived from the cleavage of the ERVK *gag-pol* polyprotein. The prime limiting factor for the identification of ERVK IN in human tissue samples is the lack of commercially available antibodies for the detection of this viral protein.

Interestingly, genomic instability is a hallmark of several ERVK-associated human diseases, including ALS (Deng et al., 2014; Maizels, 2015), schizophrenia (Smith et al., 2010; Kushima et al., 2016) and cancers (Romanish et al., 2010; Mullins and Linnebacher, 2012). Although proposed mechanisms for DNA damage do exist for these conditions, they do not preclude the involvement of ERVK IN. Conceivably, if the ERVK IN is shown to mediate DNA damage in human disease, this pathology could potentially be averted through the use of viral IN inhibitors (Gunthard et al., 2016; Kanters et al., 2016). Concrete evidence of efficacy against ERVK-driven genomic instability would be a landmark step toward developing viable therapeutic options for the treatment of these diseases.

Moreover, certain genetic backgrounds may be more susceptible to ERVK IN-driven DNA damage. STC disassembly is required for DNA damage response proteins to access lesion sites (reviewed in Lesbats et al., 2016). However, while little is known about the disassembly process, there is reason to believe that this process utilizes host cell proteins to target IN for degradation through ubiquitination or phosphorylation (Mousnier et al., 2007; Suzuki and Chew, 2012). Many of the ERVK-associated diseases are characterized by impairment of ubiquitination or abrogated proteasome function, such as in schizophrenia (Rubio et al., 2013) and ALS (Chang and Monteiro, 2015; Ciechanover and Kwon, 2015). Blockade of these pathways would be consistent with a failure to degrade IN complexes and disallow DNA repair proteins from restoring lesioned areas of the genome. Similarly, direct alterations within DNA repair systems, as seen in numerous forms of cancer (Lord and Ashworth, 2012; O’Connor, 2015), may favor the buildup of DNA damage in the context of ERVK IN expression.

### Therapeutic Value of Integrase Inhibitors in ERVK-Associated Disease

It is tantalizing to imagine how currently used anti-retroviral drugs could be repurposed for the treatment of clinically challenging conditions, such as ALS and cancers. In recent years, the adoption of IN strand transfer inhibitor (InSTI) for the treatment of HIV has come to the forefront (Gunthard et al., 2016), in part due to their improved blood–brain barrier permeability as compared to protease and RT inhibitors (Varatharajan and Thomas, 2009; Meeker et al., 2014; Nightingale et al., 2014). For example, the IN inhibitor dolutegravir has been shown to cross the blood–brain barrier, resulting in therapeutic concentrations in the cerebrospinal fluid (CSF) similar to those found in plasma (Letendre et al., 2014). This highlights the potential use of IN inhibitors for arresting progression of ERVK-associated neurological conditions.

Concern surrounding the rapid appearance of drug-resistance mutations in exogenous retroviruses commonly limits the therapeutic potential of anti-retroviral compounds (Gunthard et al., 2016; Kanters et al., 2016). However, ERVK is a prisoner entrapped by the high fidelity of human genomic replication, thus limiting the appearance of drug resistance mutations and improving the therapeutic potential for IN inhibitors in ERVK-mediated disease. Future identification of candidate IN inhibitors for clinical drug trials in ERVK-associated disease will provide a substantive contribution toward the development of effective (and desperately needed) treatment options for refractory conditions, such ALS and cancers.

## Author Contributions

MT curated the ERVK IN sequences from the GRCh38 assembly. SB performed ERVK IN alignment. MT produced the ERVK IN protein model. RD conceived the study. SB, MT, SH and RD wrote the manuscript. All authors read and approved the final manuscript.

## Conflict of Interest Statement

The authors declare that the research was conducted in the absence of any commercial or financial relationships that could be construed as a potential conflict of interest.
